# Gatekeepers of the Germ Line: How Mitochondria Shape Reproductive Evolution in Metazoans

**DOI:** 10.3390/biology14121728

**Published:** 2025-12-02

**Authors:** Yu-Tong Sun, Wan-Xi Yang

**Affiliations:** The Sperm Laboratory, College of Life Sciences, Zhejiang University, Hangzhou 310058, China; 3220101343@zju.edu.cn

**Keywords:** mitochondria, gametogenesis, mitochondria evolution, mito-nuclear coevolution, Doubly Uniparental Inheritance (DUI)

## Abstract

Mitochondria play essential roles for animal reproduction, influencing not only cellular energetics but also gamete quality, inheritance and evolutionary patterns. Currently, most research still focuses on chordates or mitochondrial diseases and their impact on the health of germ cells. However, few studies focus on integrative synthesis that connect comparative morphology, inheritance mechanisms and evolutionary theory. In this review, we integrate cross-phyla evidence to explore two interconnected dimensions: the fate of mitochondria during gametogenesis and the strategy shaping their evolution. We compare mitochondrial morphology, distribution, and metabolic strategies in gametogenesis, revealing how these traits align with reproductive modes and ecological adaptations. Then we further discuss how mitochondrial genome evolution, bottleneck effects and mito-nuclear coevolution contribute to germline stability and maternal inheritance. Special attention is given to exceptional systems such as Doubly Uniparental Inheritance (DUI) in bivalves, which challenges conventional mode of strictly maternal transmission and illuminates the flexibility of mito-nuclear evolution. Altogether, these perspectives highlight mitochondria as gatekeepers and evolutionary recorders in the reproductive systems across metazoans, providing a unifying framework for future research across ecology, evolution and molecular biology.

## Simple Summary

Mitochondria are small but vital structures inside cells, providing energy for almost all life forms. They are essential for the development of gametes, and their behaviors can influence how animals reproduce and evolve. In most species, only the mother will pass her mitochondria to the offspring, but few species show exceptions where both parents contribute. This review summarizes discoveries about how mitochondria change in shape, number, structure and activity in reproductive cells across some key species and how these changes may affect fertility and heredity to adapt to the environment. It also explores how the cooperation between mitochondrial and nuclear genes has shaped the evolution of reproductive systems in different animals. By connecting studies from cell biology, genetics and evolutionary research, this work helps explain why mitochondria are not only cells’ “powerhouse”, but also key drivers in the evolution of reproduction and species diversity.

## 1. Introduction

Mitochondria are essential organelles in almost all eukaryotic cells and are often referred to as the “powerhouse of the cell”. Their size, quantity, and morphology vary significantly across taxa and cell types. For example, giant mitochondria exceeding 4 μm are found in shrew retinal cells and horsetail worm muscle, whereas axolotl sperm carry numerous small spherical mitochondria with diameters ranging from 0.15 to 0.22 μm [1,2,3]. In terms of quantity, variation ranges from a single mitochondrion in monogenean sperm to nearly 105 in human oocytes [4,5,6]. Morphological diversity is equally noteworthy, including rod-shaped, elliptical, irregular (jigsaw puzzle piece shapes), and even reticular structures [7,8,9,10,11]. These observations highlight mitochondria as dynamic organelles that adapt to cellular and reproductive demands, with this variability forming the basis for the profound evolutionary asymmetry observed between male and female gametes.

In addition to energy conversion through ATP synthesis, mitochondria regulate calcium homeostasis [12] and participate in apoptosis, cell cycle regulation, signaling, and gene expression [13]. They also influence developmental processes such as embryonic axis formation and germ cell fate [14,15]. Unlike most organelles, mitochondria retain their own genome (mitochondrial DNA, mtDNA) that functions in close collaboration with the nuclear genome [16,17]. The bacterial origin of mitochondria is widely explained by the endosymbiotic theory, which assumes that an α-proteobacterial ancestor was engulfed by an archaeal host about 1.5 billion years ago [16]. Although strongly supported [17], this view has been refined by alternative models that highlight contributions from non-α-proteobacterial sources and anaerobic organelles [18,19].

A remarkable feature of mitochondria is their nearly universal maternal inheritance [20,21]. Paternal mitochondria delivered at fertilization are typically eliminated, preventing the inheritance of accumulated somatic mtDNA damage and subsequent genomic conflict [22]. This uniparental transmission imposes strong selective pressures within the reproductive system: during oogenesis, mitochondrial numbers expand dramatically, and quality-control mechanisms ensure preferential transmission of functional genomes [23]. The “mitochondrial bottleneck,” involving a transient reduction and amplification of mtDNA copy number, further enhances purifying selection [24]. It has even been proposed that the selection for mitochondrial quality is the primary evolutionary pressure that explains many long-puzzling features of metazoan germline evolution, including the widespread adoption of extreme oogamy, follicular atresia, and the emergence of early germline sequestration in active bilaterians [23].

Exceptions to strict maternal inheritance—such as Doubly Uniparental Inheritance (DUI) in bivalves—provide unique opportunities to study alternative transmission strategies [25,26,27]. These cases highlight the evolutionary flexibility of mitochondrial inheritance and may inform how mitochondrial behavior coevolves with germline development.

Together, these perspectives raise central questions: How do mitochondria influence gametogenesis and reproductive strategies across taxa? What mechanisms underlie their evolutionary plasticity in the germline? How do mitochondrial and nuclear genomes coevolve to maintain compatibility? And what can exceptions like DUI reveal about selective pressures shaping inheritance systems? Addressing these issues will deepen our understanding of mitochondrial roles in reproduction and their broader significance in the evolution of germline systems and sexual reproduction.

## 2. Mitochondria Fate in Gametogenesis

### 2.1. Cross-Taxa Variation in Mitochondrial Number, Distribution, and Morphology in Gametes

Mitochondria are dynamic organelles whose number, distribution, and ultrastructure vary markedly across taxa and gamete types. The vast scope of these differences across metazoan phyla is summarized in Table 1. These variations reflect the differential selective pressures imposed by maternal inheritance and the necessity for robust mitochondrial quality control to safeguard subsequent embryonic development.

#### 2.1.1. Mitochondrial Quantity Variations

Oocytes generally contain extraordinarily large mitochondrial populations, often reaching 10^5^–10^6^ per cell, as reported in annelids, arthropods, amphibians, and mammals [6,15,28,29,30,31,32]. This abundance serves as a critical mitochondrial quality control strategy, providing a large reserve of energy for embryogenesis and buffering the developing embryo against the accumulation of deleterious mtDNA mutations through a dilution effect. In contrast, sperm contain far fewer mitochondria, ranging from a single organelle in some flatworms [4] to several thousand in axolotls and birds [3,33]. Mammalian sperm usually carry fewer than 100 [12]. This asymmetry highlights the oocyte’s role as the sole mitochondrial contributor to the embryo, while sperm mitochondria are largely transient, serving motility before being eliminated post-fertilization.

#### 2.1.2. Spatial Distribution of Mitochondria

Mitochondrial positioning within germ cells also varies across lineages. In oocytes, mitochondria often shift from localized clusters to dispersed but uneven patterns during maturation. The precise relocation and even distribution of mitochondria within the cytoplasm of the mature oocyte is considered critical for high developmental competence, ensuring that each blastomere receives sufficient functional organelles post-cleavage [34]. In many species, they preferentially accumulate at the vegetal hemisphere or around the nucleus [35,36]. A conserved feature across taxa is the Balbiani body (also called the mitochondrial cloud), an aggregate of mitochondria and germ plasm components observed in insects, amphibians, birds, and mammals [30,31,37]. (To avoid conceptual ambiguity, we distinguish the two terms as follows: the Balbiani body refers to the entire multi-organelle structure—including mitochondria, Golgi elements, ER, and germ plasm components—whereas the mitochondrial cloud describes specifically the mitochondria-enriched core region.) Although historically used interchangeably, the Balbiani body encompasses a broader set of determinants and plays key roles in organelle partitioning, mitochondrial selection, and germline specification [38]. By contrast, sperm mitochondria are compactly arranged in the midpiece, often spirally coiled around the axoneme, optimizing ATP delivery for motility [39,40,41].

#### 2.1.3. Mitochondrial Ultrastructure and Morphological Remodeling During Gametogenesis

**Figure 1:**
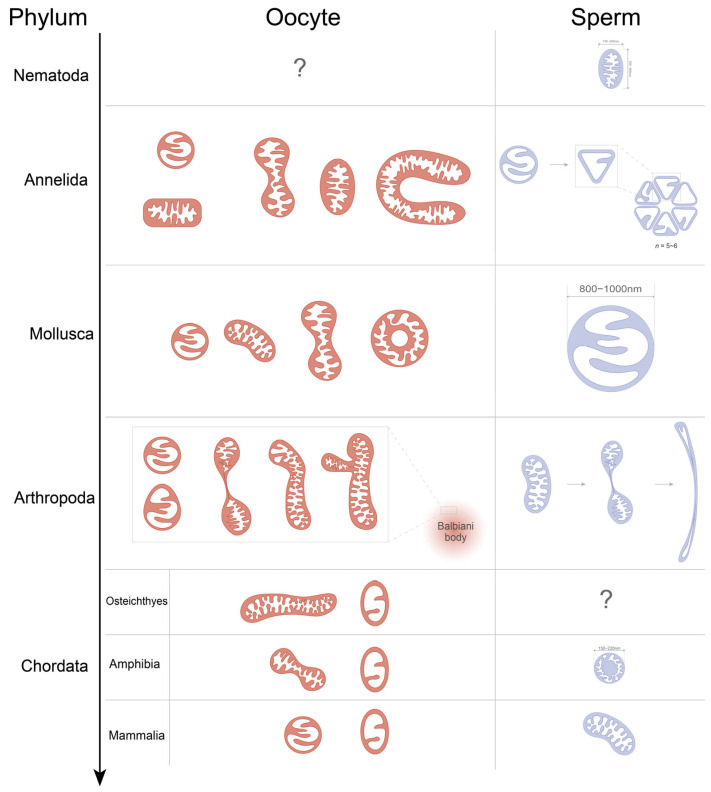
Schematic diagram of the morphology and structure of mitochondria in oocytes and/or sperm cells from different animal phyla (almost all based on electron microscopy images). Length, width, and diameter are annotated for documented values [3,26,44]; if not annotated, the size is unknown. “?” indicates that no relevant literature has been found on the physical characteristics of mitochondria. Mitochondria are highly dynamic organelles, undergoing multiple morphological and microstructural changes, particularly during embryogenesis. Mitochondrial cristae can be broadly categorized into three types: orthodox (characterized by a clear internal structure), condensed (featuring a highly dense matrix and a tightly packed arrangement of cristae), and arched cristae (located peripherally) [3,36,42,47,48]. Mitochondria and other material can also accumulate near the nucleus in arthropods and amphibians, often referred to as “mitochondrial cloud” or “Balbiani body” [30,37,38].

Oocyte mitochondria typically display rounded or elongated morphologies with defined cristae, whose density will increase during maturation [28,31]. However, mitochondria of some certain taxa exhibit unusual forms—such as annular (“donut-shaped”) or fused-cristae mitochondria in mollusks and sturgeons [36,42], as shown in Figure 1—likely reflecting species-specific adaptations to metabolic needs. The shift in cristae morphology, such as the change from condensed to the sparse, peripheral arched cristae observed during oocyte maturation, reflects a crucial transition in metabolic state and function—from biogenesis to quiescence—that minimizes ROS production and preserves mtDNA integrity. In sperm, mitochondria also undergo remodeling: for instance, mitochondria in earthworms and locusts show wedge-shaped and crescent-like structures, respectively, as shown in Figure 1 [39,40,43]. These modifications generally correlate with the high energy demands of sperm motility.

**Table 1:** Cross-Phyletic Comparison of Mitochondrial Number, Distribution, and Morphology in Animal Gametes.

Taxa	Gamete	Species	Mitochondrial Number	Distribution	Morphological Features
Platyhelminthes	Sperm	*Pseudodactylogyrus* sp. [4]	1	/	/
Nematoda	Sperm	*Admirandus multicavus* [44]	>50 per cross-section	Scattered in cytoplasm between membranous organelles (MOs)	Oval; ~0.3–0.4 µm long, 0.1–0.2 µm wide
Annelida	Oocyte	*Enchytraeus albidus* [28]	~10^5^	Dispersed among yolk, traversed by annular tubes	Round to elongated, sometimes branched, with cristae
		*Insulodrilus bifidus* [35]	Few in early stages; markedly increase at vitellogenesis	Cytoplasm, especially periphery	Early: round/oval; Later: elongated/rod-like
	Sperm	*Lumbricus terrestris* [39]	6	Posterior pole of nucleus, later midpiece	From round → wedge-like; outer membranes fuse into hexagonal frame; reduced cristae
		*Isochaetides arenarius* [43]	Eusperm: 5; Parasperm: 2–3 (rarely 4)	Midpiece	Eusperm: cylindrical-fan shaped; Parasperm: oval, sector-like
Mollusca	Oocyte	*Ilyanassa obsoleta* [36]	/	Pre-vitellogenic: clustered at vegetal pole near follicle cells; Vitellogenic: distributed in both poles, more at vegetal pole	Pre-vitellogenic: diverse (round, elongated, dumbbell, donut-shaped), with cristae and dense granules; Vitellogenic: mainly round, occasional fused forms resembling autophagosomes
	Sperm	*Pitar rudis* [5]	4 (10% with 5)	Midpiece	Typical clustered midpiece mitochondria
		*Chamelea gallina* [5]	4	Midpiece	Similar to *P. rudis*
		*Meretrix* sp. [45]	5	Arranged around centriole complex	Densely packed, well-developed cristae
		*Ruditapes philippinarum* [26]	/	Aggregation or dispersion linked to embryo sex	Sperm mitochondrial diameter 800–1000 nm; oocyte mitochondrial diameter ~600 nm (few >500 nm)
Arthropoda	Oocyte	*Meconema meridionale* [37]	/	Bouquet stage: mitochondrial network with nuage; later fragmented into smaller networks, finally single mitochondria	Network → micro-networks → single mitochondria
	Sperm	*Melanoplus differentialis* [40]	/	Midpiece/flagellum	Large mitochondria elongate into filaments, C- or crescent-shaped around nucleus
Osteichthyes	Oocyte	*Polyodon spathula and Acipenser gueldenstaedtii* [42]	/	Cytoplasm of dictyotene and previtellogenic oocytes	Two types: (1) elongated with well-developed cristae, often near nucleus and nuage; (2) spherical with deformed/fused cristae, randomly distributed, sometimes with lipid-like inclusions; deformed mitochondria degenerate and fuse with lipid droplets
Amphibia	Oocyte	*Xenopus laevis* [29,30,31,32]	>5 × 10^5^ when oocyte diameter ~300 µm	Uneven distribution in ooplasm	Prominent Balbiani body
	Sperm	*Ambystoma mexicanum* [3]	3200–4000	Midpiece, tightly packed in semicircular sheet covering dense core	Very small (0.15–0.22 µm), spherical, with outer and inner membranes and round cristae; contain electron-dense vesicles
Aves	Oocyte	*Coturnix japonica* [30]	/	Two groups: one forms a “crown” around germinal vesicle, another migrates to vegetal pole (future germ cells)	Typical oocyte mitochondria
	Sperm	*Coturnix japonica* [33]	>1400	Midpiece, helically arranged around axoneme, covering 64–74% of sperm length (160–170 µm)	Double-membrane; cristae parallel to outer membrane
Mammals	Oocyte	*Homo sapiens* [6,15,31]	~10^5^	Uniform or perinuclear clustering; denser in inner cytoplasm	Round, sparse arched cristae, contacts with smooth ER
		*Mus musculus* [31]	Increase from GV → MI → MII	GV: dispersed; MI: clustered in inner cytoplasm; MII: larger clusters inside cytoplasm	Round/oval, few cristae, low metabolic activity
	Sperm	Mammals (general) [41]	~100 mtDNA copies	Midpiece, spiral arrangement	Typical helical sheath
		*Meriones unguiculatus* [46]	/	Early: dispersed in cytoplasm; Later: spiral around midpiece	Elongated, helically arranged mitochondria

### 2.2. Mitochondrial Functions During Gametogenesis

Mitochondria are central regulators of gametogenesis, supporting germ cell maturation and quality through energy metabolism, signaling, apoptosis, epigenetic regulation, and genome transmission. Collectively, these processes constitute the mitochondrial quality control system, which acts as a “gatekeeper” to ensure the fitness of the succeeding generation.

#### 2.2.1. Energy Metabolism

Mitochondria provide ATP critical for gamete development and function. During spermatogenesis, cells undergo metabolic reprogramming: spermatogonia rely on glycolysis, differentiating spermatocytes gradually shift toward oxidative phosphorylation (OXPHOS), while mature sperm often revert to glycolysis-dominated metabolism, though species-specific strategies exist [49,50]. Structurally, sperm mitochondria form a sheath tightly packed in the midpiece, optimized for motility and fertilization [41]. In oocytes, mitochondria emphasize stability and fidelity. Although OXPHOS is dominant, oocytes preferentially metabolize pyruvate, and mitochondria remain partly quiescent to minimize ROS-induced mtDNA damage [49,50,51]. A high mitochondrial number ensures ATP reserves that dilute mutant mtDNA during cleavage and support energy-intensive processes like spindle assembly, meiosis, and fertilization. Thus, mitochondrial abundance and ATP output are tightly correlated with oocyte quality and developmental competence [21].

#### 2.2.2. Redox Balance and ROS Signaling

Mitochondria are major sources of reactive oxygen species (ROS), which act as double-edged regulators. At physiological levels, ROS function as signaling molecules required for sperm capacitation, acrosome reaction, and oocyte maturation [50,51,52]. Excess ROS, however, causes lipid peroxidation, DNA fragmentation, and apoptosis, impairing gamete quality. Antioxidant systems can buffer these effects, with disruptions leading to infertility or ovarian dysfunction [49]. Thus, maintaining ROS homeostasis is central to both sperm function and oocyte viability. The oocyte’s strategy of metabolic restraint is itself a mitochondrial quality control mechanism evolved to minimize ROS production and safeguard the integrity of the mtDNA template for subsequent generations.

#### 2.2.3. Apoptosis and Germ Cell Selection

Mitochondria govern intrinsic apoptosis via cytochrome c release and caspase activation, eliminating defective germ cells and ensuring reproductive quality [12]. In spermatogenesis, mitochondrial apoptotic pathways remove impaired spermatogonia; defects in pro-apoptotic regulators such as BAX disrupt clearance, leading to infertility or tumorigenesis [49]. In females, germ cell selection during fetal oogenesis and folliculogenesis involves massive waves of apoptosis, refining the oocyte pool [24,53]. In fact, this widespread culling, termed follicular atresia, functions as a large-scale mitochondrial quality control mechanism, maximizing the segregational variance in mitochondrial quality among surviving oocytes to ensure only the fittest pools are transmitted [23]. The mitochondrial protease LONP1 and germline-specific cytochrome c isoforms exemplify critical molecular hubs for this quality control. By efficiently clearing compromised proteins, LONP1 protects the integrity of the OXPHOS complexes and maintains the high membrane potential required for functional survival, thereby directly supporting gamete quality and developmental potential [50,54].

#### 2.2.4. Epigenetic Regulation

Mitochondrial metabolism supplies key intermediates—acetyl coenzyme A (acetyl-CoA), α-ketoglutarate, and S-adenosylmethionine—that shape histone modifications, DNA methylation, and genomic imprinting [24,49,51]. Acetyl-CoA serves as the essential substrate for histone acetylation (e.g., H3K27ac), while α-ketoglutarate fuels TET-mediated DNA demethylation, and SAM provides the methyl donor required for DNA and histone methyltransferases. These metabolites directly influence the activity of epigenetic regulators such as the NAD^+^-dependent deacetylase SIRT1 and the maintenance methyltransferase DNMT1, whose functions are highly sensitive to mitochondrial metabolic state [51]. Age-related mitochondrial dysfunction lowers NAD^+^ availability and SAM synthesis, leading to reduced SIRT1 and DNMT1 activity, aberrant histone acetylation, and instability of DNA methylation at imprinted loci (e.g., Igf2, H19). Such disruptions alter the expression of genes critical for oocyte competence and Zygotic Genome Activation (ZGA), including pluripotency regulators (OCT4, SOX2) and factors coordinating OXPHOS–nuclear transcription programs [51]. Thus, mitochondria can act as metabolic-epigenetic hubs that couple energy state with germline programming.

#### 2.2.5. Mitochondrial DNA Transmission and the Bottleneck Effect

A hallmark of gametogenesis is the strict maternal inheritance of mtDNA, enforced by the elimination of sperm-derived mitochondria after fertilization [21]. Complementing this, the mitochondrial bottleneck—characterized by a transient reduction and amplification of mtDNA copy number—limits heteroplasmy and promotes purifying selection [23]. Quality control mechanisms also act within oocytes: in *Drosophila*, PINK1-mediated suppression of defective mitochondria prevents their propagation [55], while mammalian oocytes exclude deleterious mtDNA variants [49]. TFAM degradation further ensures paternal mtDNA clearance [56]. These processes suggest that safeguarding mitochondrial integrity was a key evolutionary driver of germline sequestration [23]. Notably, exceptions such as DUI in bivalves [26,57] reveal the plasticity of inheritance strategies, where both F- and M-type mtDNA persist during early development, highlighting alternative evolutionary solutions to germline mitochondrial quality control.

### 2.3. Evolution of Mitochondrial Bioenergetics and Its Link to Reproductive Strategies

Mitochondrial metabolism is tightly coupled to reproductive biology. Understanding this divergence is crucial as it reveals the differential selective pressures imposed by maternal inheritance on the two gamete types. Variations in mitochondrial bioenergetics across taxa not only sustain gamete function but also shape reproductive strategies in response to ecological pressures [58,59,60,61]. This section addresses the evolutionary trade-off: genomic fidelity versus maximal energetic function.

#### 2.3.1. Evolutionary Plasticity of Mitochondrial Metabolism

Cells generate ATP mainly through glycolysis and oxidative phosphorylation (OXPHOS). Glycolysis is rapid and oxygen-independent but inefficient, while OXPHOS is more productive but requires oxygen [59]. Some organisms illustrate extreme adaptations: the cnidarian parasite *Henneguya salminicola* has completely lost its mitochondrial genome, relying on anaerobic metabolism [62], while its close relative *Myxobolus squamalis* retains respiration. In hypoxic marine environments, sponges and nematodes perform anaerobic respiration using fumarate as an electron acceptor [59,63]. Mitochondrial derivatives such as hydrogenosomes and mitosomes in diverse eukaryotes further illustrate the organelle’s plasticity [59]. In aquatic taxa like mollusks and fish, mitochondria flexibly use amino acids, fatty acids, or ketone bodies depending on oxygen levels and substrate availability [63]. These examples illustrate that mitochondrial bioenergetics are evolutionarily malleable, providing a toolkit for species to adapt to diverse ecological constraints.

#### 2.3.2. Divergent Bioenergetic Strategies in Gametes

Oocytes and sperm adopt contrasting energy strategies. Oocytes are metabolically restrained and prioritize mtDNA stability, often depending on pyruvate and lactate supplied by cumulus cells to minimize ROS damage [59]. During maturation, however, OXPHOS becomes increasingly important to accumulate reserves for cleavage and blastocyst formation [64]. In contrast, sperm are specialized for motility and rely on mitochondria concentrated in the midpiece to power flagellar movement via OXPHOS. Yet they remain metabolically flexible: porcine sperm are glycolysis-dependent, while bovine sperm can switch to oxidative pathways under certain conditions [60,65]. High glycolytic activity often correlates with motility and fertilization capacity [65]. This divergence illustrates a clear evolutionary trade-off: oocytes prioritize genomic integrity and developmental competence by favoring a lower ROS state, while sperms maximize energetic efficiency for fertilization success. This metabolic asymmetry is also a direct result of differences in mitochondrial DNA copy number: the lower copy number in sperm (<100 copies) makes them extremely sensitive to mitochondrial DNA mutations and oxidative phosphorylation dysfunction. Conversely, the higher copy number in oocytes buffers female lineages from the effects of the same mutations [66].

#### 2.3.3. Species-Specific Metabolic Strategies and Environmental Adaptation

Across taxa, reproductive metabolism reflects both species traits and ecotope. Metabolic constraints can dictate the evolution of life-history traits. For example, insects like *Drosophila* rely on glycolysis under hypoxic niches [61], while the “Warburg effect” (aerobic glycolysis) seen in some taxa allows for rapid ATP production under resource limitations [67,68]. Crucially, unique inheritance systems have evolved to manage these metabolic demands. In Doubly Uniparental Inheritance (DUI) bivalves, the divergence between male (M-type) and female (F-type) mitochondrial lineages is accompanied by sex-specific transcriptional patterns, suggesting that DUI may have evolved to allow sperm to maintain high metabolic rates without risking the genetic integrity of the population’s transmission lineage (F-type) [58]. Furthermore, mitochondrial metabolites act as essential precursors for epigenetic regulation during early embryogenesis [24]. Thus, the evolution of mitochondrial metabolism is not merely a cellular adaptation but a central driver of reproductive diversity and specification.

### 2.4. Challenging Maternal Mitochondrial Inheritance: The Case and Evolutionary Significance of Doubly Uniparental Inheritance (DUI)

Maternal inheritance of mitochondria is nearly universal among metazoans, ensuring genome integrity and homogeneity. However, an exceptional system—DUI—occurs in certain bivalves, where both maternal (F-type) and paternal (M-type) mitochondria are inherited in a sex-specific manner: females transmit F-type to all offspring, while males pass M-type only to male offsprings [25,27,58,69,70,71,72,73]. DUI has been reported across multiple bivalve families, including Mytilidae, Veneridae, and Unionidae [27,72], and remains the only known case of stable non-maternal inheritance in animals [71].

DUI challenges the notion that strict maternal inheritance is indispensable for mitochondrial stability, suggesting instead that inheritance rules can be more flexible and potentially adaptive. Crucially, the existence of DUI suggests that the evolutionary selection for maintaining functional mitochondrial activity and mitochondrial quality control mechanisms might be more fundamental than the selection for strict uniparental transmission. F- and M-type lineages show extreme divergence—sometimes exceeding 50% in protein-coding regions [69]—and their coexistence provides a natural model for exploring heteroplasmy, sex determination, germline development, and mitonuclear interactions [73]. In some species, such as *Arctica islandica*, M-type mtDNA even dominates somatic tissues, possibly reflecting environmental adaptation and relaxed segregation during embryogenesis [25,26]. Moreover, M-type mitochondria in the DUI system are not functionally silenced but maintain high activity (including transcriptional activity and membrane potential) within the male germline, indicating that they must actively overcome the problem of maintaining genetic information viability while producing ATP for motility [23].

**Figure 2:**
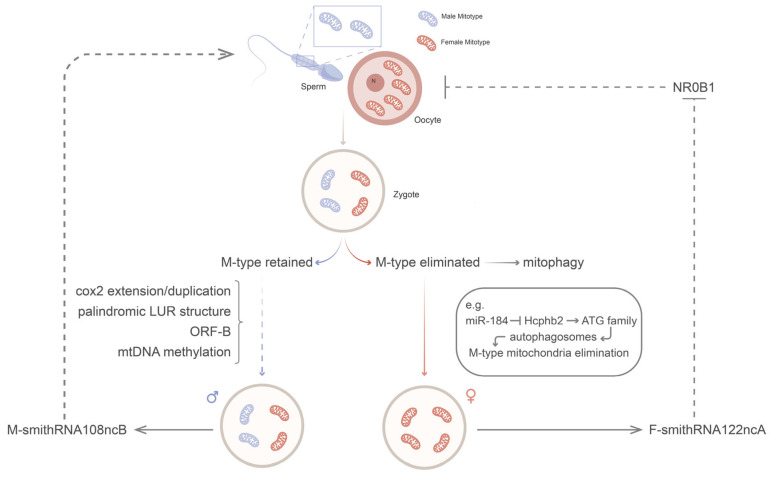
Schematic diagram of the DUI phenomenon (primarily involving mitochondria). Blue represents paternal mitochondria and the genetic pathway, while red represents maternal mitochondria and the genetic pathway. Solid lines represent actual/evidence-proven pathways and mechanisms, while dashed lines represent possible/unproven/speculated pathways and mechanisms. Sex-linked smithRNA may affect germ cells by influencing gonadal development. N: nuclear.

The persistence of two distinct mitochondrial lineages within a single species raises key evolutionary questions: how sperm-derived mitochondria are selectively maintained, how bottleneck dynamics are regulated, and what functional contributions M-type mtDNA makes to male fertility. The complex transmission and sex-specific fates of F-type and M-type mtDNA are schematically illustrated in Figure 2, highlighting the dual-lineage propagation and the potential influence of sex-linked factors. Mechanistically, the retention of M-type mitochondria may be associated with unique genomic structures or modifications found in DUI species, such as cox2 gene extension/duplication, palindromic LUR strutures, ORF-B presence, and mtDNA methylation, although the direc functional links require further validation [70,74,75]. Conversely, the selective elimination of M-type mtDNA in the female lineage is often achieved via specialized mitophagy pathways [76]. Furthermore, DUI systems offer insight into novel mito→nuclear retrograde signaling mechanisms [77]. This complex interplay of sncRNAs suggests a potent new axis for mitochondrial control over sex determination and germline programming.

Over long timescales, DUI systems offer unique opportunities to study the evolutionary plasticity of mitochondrial transmission strategies, lineage-specific adaptation, and the mito-nuclear coevolution [70,72].

The molecular basis of DUI—including sex- and tissue-specific fates of M-type mtDNA—will be elaborated further in Section 3.1.2, which also can be seen in Figure 2.

## 3. Evolutionary Strategy of Mitochondrial Inheritance and Coevolution

### 3.1. Evolution of the Mitochondrial Genome

#### 3.1.1. Mitochondrial Genome Content and Structure

The endosymbiotic theory posits that mitochondria originated from α-proteobacteria, with subsequent massive gene loss or transfer to the nucleus, leaving >98% of mitochondrial proteins encoded by nuclear genes [78]. The consequence of this ancient event is the establishment of an obligate dual-genome system where the nuclear and mitochondrial genes must maintain absolute functional synchrony to ensure the integrity of the crucial OXPHOS pathway. This highly conserved interaction is complicated by the asymmetric evolutionary pressure stemming from the mtDNA’s order of magnitude higher mutation rate compared to nuclear DNA in metazoans. In most metazoans, the mitochondrial genome is highly conserved, typically comprising 37 genes: 13 protein-coding, 2 rRNA, and 22 tRNA [58,79,80,81,82]. Nonetheless, notable exceptions exist. For example, atp8 is absent in nematodes and some sponges [80,83], though misannotation has been proposed in mussels [84]. Variability is also seen in tRNA counts, with DUI species like *Ruditapes philippinarum* containing 23–24 tRNAs [85]. Additional open reading frames (ORFs) have been reported in sponges and cnidarians [86], and some may have functional roles in reproduction, as seen with CYTB-187AA affecting mouse fertility [87].

Structurally, most mtDNA is circular and intron-free [82,88], but exceptions highlight evolutionary plasticity: (i) Genome loss: *Henneguya salminicola* retains mitochondria but has lost mtDNA entirely, reflecting adaptation to anaerobic metabolism [62]; (ii) Linear genomes: found in cnidarians and sponges, maintained by specialized replication mechanisms [89,90,91]; (iii) Introns: reported in sponges and cnidarians, pointing to horizontal transfer events [92]. These structural and content variations reflect species-specific adaptations to specialized metabolic niches and the variable evolutionary tension between the two genomes, underscoring the mtDNA’s role as an adapting subunit in the mito-nuclear coevolutionary framework.

#### 3.1.2. Exceptions: DUI Systems with F-Type and M-Type mtDNA

DUI in bivalves represents a striking deviation from strict maternal transmission. Females transmit F-type mtDNA to all offspring, while males carry both F-type and M-type, passing M-type only to male offsprings [27]. This system likely originated from modifications in paternal mtDNA elimination, coupled with sex-determination processes tied to M-type retention. Phylogenetic analyses suggest a single origin followed by recombination, explaining non-monophyly of M genomes [72]. Functionally, DUI sperm rely on active OXPHOS, with recombination potentially facilitating mito-nuclear coevolution and reducing mutational load [71].

The DUI system provides a unique model to study how selection for functional mitochondrial quality control drives the evolution of specialized genomic structures. As shown in Figure 2, several molecular features are candidate hallmarks of DUI. In *Musculista senhousia*, cox2 duplications and palindromic structure of the Large Unassigned Region are unique to M-type genomes [74]. Novel ORFs, such as F-ORFs (ORFs found in the F-mtDNA) and ORF-B, are conserved across DUI taxa and hypothesized to protect M-type mitochondria from degradation [79,93]. Epigenetic control also plays a role: mtDNA methylation may shield paternal mtDNA from elimination [75]. In clams, the PHB2–miR-184 axis regulates autophagic clearance, with knockdown experiments confirming its role in M-type persistence or elimination [76].

Beyond coding sequences, mitochondrial small RNAs (smithRNAs) influence gonad development, with F-type smithRNAs promoting ovarian pathways and M-type smithRNAs potentially regulating spermatogenesis [77]. Cytological evidence further reveals sex-specific mitochondrial dynamics: in *Mytilus* and *Ruditapes*, sperm mitochondria aggregate in males but disperse and are degraded in females, suggesting conserved patterns of selective elimination [26,94]. Mechanistically, degradation may occur via delayed ubiquitin-mediated pathways or replication suppression combined with dilution.

Ecological adaptations add further complexity. In *Arctica islandica*, high-latitude populations sometimes exhibit somatic dominance of M-type mitochondria, a potential cold-environment adaptation [25]. Population-level studies in clams also reveal paternal bottlenecks, with high variability of M-type mtDNA across tissues [85]. Crucially, DUI lineages support the universality of the mito-nuclear coevolutionary principle: evolutionary rate covariation analyses indicate both F- and M-type genomes coevolve tightly with nuclear OXPHOS genes, demonstrating that OXPHOS compatibility is fiercely selected across all inheritance systems [95].

Origin models liken DUI to cytoplasmic male sterility (CMS) in plants: feminizing F-ORFs may have caused male sterility in ancestral lineages, later countered by nuclear restorer genes (e.g., M-ORFs or cox2 extensions) [96]. While suggestive parallels exist, direct functional validation remains lacking.

In summary, DUI represents not only a peculiar inheritance pattern but also a unique window into mito-nuclear coevolution in the contexts of sex determination, energy metabolism, and epigenetic regulation. However, despite numerous hypotheses and fragmented evidence, its molecular mechanisms and evolutionary significance remain far from fully understood, warranting further integrative studies that combine functional experiments with cross-species comparisons.

### 3.2. Maternal Inheritance and the Mitochondrial Bottleneck

Maternal inheritance, achieved through stringent clearance of paternal mitochondria, constitutes the first barrier of germline quality control. However, this alone cannot explain the pronounced variability in heteroplasmy among offspring from the same mother. This paradox points to a second filter—the mitochondrial genetic bottleneck—during which mtDNA copy number transiently contracts and subsequently re-expands during oogenesis and early embryogenesis, reshaping allelic frequencies and preferentially transmitting healthier mitochondrial genomes [97,98,99].

**Figure 3:**
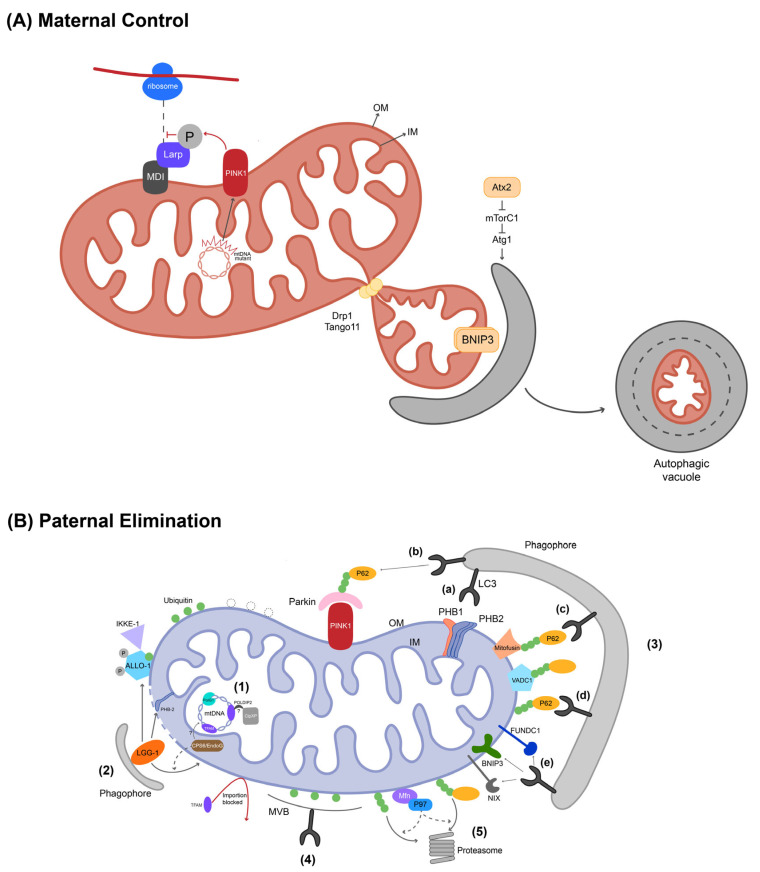
Possible mechanisms of maternal inheritance and bottleneck effects. (**A**) Maternal mitochondrial quality control: mtDNA copy number first contracts and then expands, enabling selective removal of defective mitochondria via PINK1–Larp-mediated suppression of replication and programmed germline mitophagy (PGM), which involves BNIP3, Drp1/Tango11, and Atg1-dependent autophagic activation. Mitochondrial clustering structures such as the Balbiani body may further facilitate quality-based selection. (**B**) Paternal mitochondria and mitochondrial genome elimination: (1) Paternal mtDNA clearance involves EndoG (CPS-6), PolG1, and POLDIP2 (*D. melanogaster*), or TFAM blockade (mammals). (2) In *C. elegans*, CPS-6 (EndoG) degrades mtDNA after loss of membrane potential; ALLO-1 and PHB-2 recruit LGG-1/2 to form allophagosomes. (3) Degradation pathways: PHB-2 can bind to LC3 to mediate recognition and clearance (a); FNDC-1 (FUNDC1 homolog) selectively marks paternal mitochondria (e); PINK1/Parkin promotes ubiquitination and recruits p62 and LC3 (b); VDAC1 and mitofusin serve as ubiquitination targets (c); p62 recognizes ubiquitinated cargo and interacts with LC3 (d); NIX and BNIP3 achieve autophagy through non-ubiquitin-dependent pathways (e). (4) *D. melanogaster* sperm mitochondria are degraded through a phagocytosis-like pathway. (5) The ubiquitin-proteasome system (UPS) and the protein dislocase VCP/p97 contribute to elimination (mammals). P: phosphorylation modification; OM: outer membrane; IM: inner membrane; “?” indicates the mechanism remains unclear.

Across animal phyla, the paternal barrier follows a broadly conserved “two-stage program.” In many species, mtDNA is already eliminated during spermatogenesis, such that sperm delivered at fertilization contribute virtually no intact copies. In mammals, this is linked to a testis-specific isoform of TFAM that cannot be imported into mitochondria, leaving mature sperm with negligible mtDNA [20,100]. After fertilization, paternal mitochondria are removed by maternal quality-control systems. These highly conserved mechanisms rely heavily on the autophagy–lysosome and ubiquitin–proteasome pathways. In *C. elegans*, for example, sperm mitochondria lose membrane potential, their genomes are degraded by the nuclease CPS-6 (EndoG), and autophagy proteins such as ALLO-1 and PHB-2 recruit LGG-1 to package the organelles into allophagosomes [101,102,103]. These processes are detailed in Figure 3B, which illustrates various mechanisms including ubiquitin-mediated tagging (p62 and VCP/p97), non-ubiquitin-dependent mitophagy (NIX/BNIP3), and specialized degradation pathways involving FNDC-1 and TFAM blockade. Comparable mechanisms, though employing distinct adaptors, are also evident in flies and mammals, underscoring a shared reliance on autophagy–lysosome and ubiquitin–proteasome pathways [104,105]. Rare cases of paternal leakage highlight that maternal inheritance is not automatic, but actively enforced. And comparative analyses reveal a backdrop of mechanistic convergence. Yet deviations occur in some lineages: bivalves and certain insects can retain paternal mitochondria, often linked to suppressors of ubiquitin tagging or autophagic engagement. Even in these cases, however, crosstalk between the ubiquitin-proteasome system and autophagy, mediated by proteins like p62 and VCP/p97, provides multiple safeguards against uncontrolled inheritance of paternal genomes [104,105].

The genetic bottleneck itself is increasingly recognized as an active, quality-linked process rather than a stochastic contraction. This process, schematically shown in Figure 3A (Mitochondrial Quality Control), involves the selective elimination of defective maternal mitochondria coupled with the biased amplification of functional ones. In *Drosophila*, inhibition of mTORC1 at meiotic entry initiates programmed germline mitophagy (PGM), in which defective mitochondria recruit the autophagy receptor BNIP3 and undergo Drp1/Tango11-mediated fission, while Atx2 and other negative regulators activate the Atg1 complex to generate autophagic structures [106,107]. Concurrently, damaged mitochondrial genomes are selectively prevented from replicating: PINK1 phosphorylates Larp to block local protein synthesis on the mitochondrial surface, thereby restricting mtDNA replication within defective organelles [55]. These mechanisms couple selective elimination with biased amplification, ensuring that expansion after contraction favors functional genomes.

Spatial organization of oocytes further contributes to bottleneck selectivity. In amphibians and fish, the Balbiani body aggregates mitochondria and RNAs into a compartment where high-quality organelles are preferentially retained, while defective ones may be targeted by autophagic structures [37,108]. This suggests that amplification and clearance are coupled within the same subcellular context, making spatial pre-sorting an additional layer of quality control.

Environmental cues also modulate bottleneck depth and timing. In a mouse ESC–PGCLC model, hypoxia reduces mtDNA copy number by limiting replicating foci, thereby enhancing heteroplasmy segregation, whereas hyperoxia blocks this contraction [98]. Single-cell transcriptomics suggest that oxygen tension directly regulates replication genes such as *Mthfd2* and *Mgme1*, linking developmental environment to mitochondrial inheritance fidelity [98].

### 3.3. Mito-Nuclear Coevolution in the Reproductive Dimension

**Figure 4:**
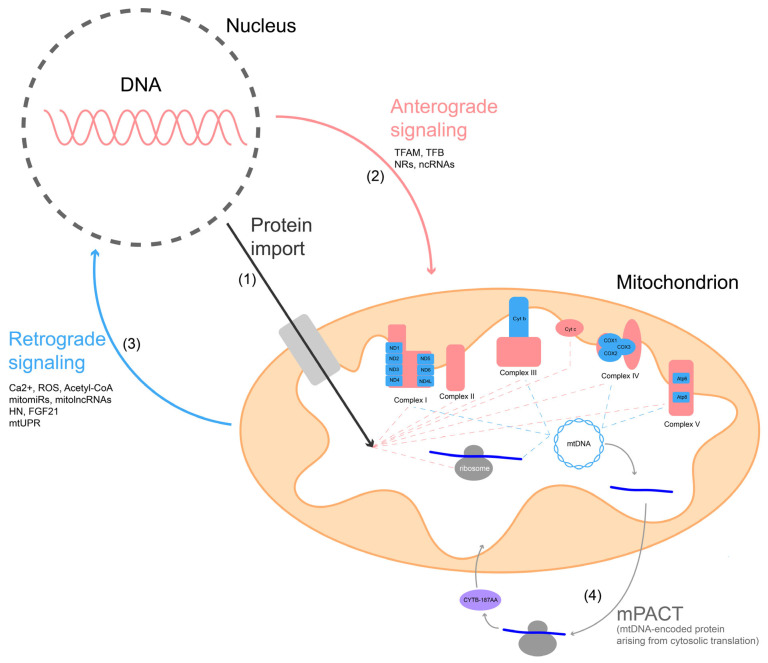
Interactions between the mitochondrion and nucleus. (1) The proteins encoded by the nuclear genome that enter the mitochondria (the red part of the complex) are essential for the construction of all complexes of the electron transport chain (red dashed lines). The proteins encoded by the mitochondrial genome (the blue part of the complex) are very important for Complex I, III, IV, and V (blue dashed lines). In addition, the proteins encoded by both genomes are also important for the ribosomes in the mitochondria. (2) Anterograde (nucleus to mitochondria) signaling pathway regulates mitochondrial gene expression through the nuclear-encoded transcription factors (TFs, TFAM and TFB that bind the mtDNA), nuclear receptors (NRs) and ncRNAs. (3) Retrograde (mitochondria-to-nucleus) signaling pathway enables the nucleus to sense mitochondrial health through retrograde signals such as Ca^2+^, ROS, acetyl coenzyme A (Acetyl-CoA), mitochondria microRNAs (mitomiRs), mitochondria long non-coding RNAs (mitolncRNAs), humanin (HN), fibroblast growth factor 21 (FGF21) and mitochondrial unfolded protein response (mtUPR). (4) Cytoplasmic translation of mitochondrial mRNA (mPACT) can generate new peptides (such as CYTB-187AA), which regulate SLC25A3 and affect ATP production.

Mito-nuclear coevolution describes the reciprocal adjustment between mtDNA and nuclear genes in order to maintain the integrity of OXPHOS complexes and mitochondrial translation. Cross-species mitochondrial genome swaps that impair Complex I activity demonstrate that divergent mutation rates in the two genomes can create incompatibilities, and that maintaining compatibility is critical for organismal fitness. This requirement is especially acute during gametogenesis and early embryogenesis, when the mitochondrial population transmitted to the next generation is determined [100,101]. In this sense, mito–nuclear compatibility itself acts as a reproductive gatekeeper, filtering which mitochondrial lineages are eligible for transmission and thereby shaping germline continuity. Because animal mtDNA evolves rapidly and is strictly maternal, nuclear genes must continually adjust to preserve protein–protein and RNA–protein interactions. This continual adjustment often takes the form of compensatory evolution, where the nuclear genome evolves “restorer alleles” to rescue or buffer defects caused by mtDNA mutations [109,110]. This process links directly to fitness and may contribute to reproductive isolation. In general, mito-nuclear coevolution operates through three main interfaces, as schematically illustrated in Figure 4: (1) Protein–Protein interactions within the OXPHOS complexes; (2) Protein–RNA interactions (mainly involving the mitochondrial ribosome); and (3) Protein–DNA interactions (at the mitochondrial replication and transcription machineries). Efficient respiration relies on a good matching of mitochondrial and nuclear alleles [101,110,111].

During oogenesis, mitochondrial quality control is tightly coupled to reproductive programs. In *Drosophila*, programmed germline mitophagy triggered by mTORC1 inhibition coordinates with meiosis to eliminate defective organelles, thereby ensuring a founder pool compatible with the nuclear background [97]. In mammals, the mitochondrial protease LONP1 prevents inappropriate nuclear translocation of AIFM1, safeguarding oocyte viability [54]. In *C. elegans*, CCR4-NOT–mediated “storage bodies” regulate nuclear-encoded mitochondrial proteins, and MTERF proteins bind mtDNA to control transcription and replication, illustrating how the nuclear genome modulates mitochondrial function [112,113]. Similarly, transcriptional regulators such as PGC-1α, NRF1/2, and YY1 integrate retrograde signaling to coordinate mitochondrial biogenesis in germ cells [78,111]. Figure 4 details this retrograde signaling, where mitochondria sense their health status via signals such as ROS, Ca^2+^, Acetyl-CoA, and mitochondrial small RNAs (mitomiRs, mitolncRNAs) to modulate nuclear gene expression [114]. Together, these oogenic pathways function as gatekeeping checkpoints: only mitochondria that pass compatibility and quality thresholds are allowed to enter the maternal transmission bottleneck.

In spermatogenesis, paternal mitochondria are destined for elimination, narrowing the window for direct mito-nuclear interaction. Nevertheless, morphological studies show clustering of mitochondria near the nuclear envelope in spermatocytes, suggesting transient exchange. Under maternal inheritance, mtDNA variants deleterious to males but neutral in females may accumulate; nuclear “restorer alleles” in the testis are then favored to offset these male-specific costs [115]. This asymmetry reinforces the gatekeeping role of mitochondrial inheritance, whereby only maternal-compatible mitochondrial populations contribute to the next generation.

Whether fertilization and early embryogenesis directly test mito-nuclear compatibility remains uncertain. One example is cytosolic translation of mitochondrial mRNAs (mPACT) in mammals, shown in Figure 4, producing peptides such as CYTB-187AA that modulate nuclear-encoded proteins and female fertility [87]. By contrast, retrograde stress pathways are well established in somatic contexts but lack direct reproductive evidence [101]. Likewise, mitochondrial RNA processing and small RNAs (smithRNAs) can influence nuclear gene expression [114,116], though their post-fertilization roles remain to be clarified. Even so, these early embryonic stages likely represent a final compatibility checkpoint, eliminating defective mito-nuclear combinations before lineage establishment.

DUI bivalves provide a distinctive model in which both F-type and M-type mtDNA must each coordinate with the same nuclear genome. Genomic features such as cox2 extensions and sex-linked ORFs have been proposed as mediators of mitochondrial recognition or function during male development [70,96]. Functional assays in *Hyriopsis cumingii* show that the PHB2–miR-184 axis controls female clearance of M-type mitochondria, illustrating nuclear regulation of mtDNA fate [76]. Expression analyses reveal tissue specificity of F-type and M-type transcripts, while recombination between lineages may offset mutational load and sustain mito-nuclear compatibility [71,72]. Population genomic signals of dN/dS suggest that compatibility is actively maintained, not merely the product of relaxed selection [117]. Interestingly, in *Arctica islandica*, persistence of M-type mitochondria in somatic tissues of high-latitude populations indicates that environmental factors can influence optimal compatibility solutions [25]. Thus, DUI systems highlight a dual-lineage scenario in which gatekeeping is exercised twice—once for each mtDNA type—demonstrating the universality of compatibility enforcement.

Overall, mito-nuclear coevolution in reproduction operates through three main interfaces: protein–protein contacts in OXPHOS complexes, protein–RNA interactions in translation, and nuclear factors binding mtDNA. Disruption at these levels can increase disease risk, impair fertility, or under certain conditions drive speciation [113]. With mtDNA evolving rapidly and maternal inheritance shaping selective pressures, gametogenesis and early embryogenesis represent privileged stages for testing mito-nuclear compatibility and for understanding how coevolution contributes to both fitness and reproductive isolation.

## 4. Conclusions

Mitochondria are central to germline biology, influencing the quality of gametes through their number, distribution, ultrastructure, and genetic integrity. Maternal inheritance and the mitochondrial bottleneck provide complementary quality-control filters, while non-standard systems such as DUI demonstrate that inheritance pathways are not fixed but evolutionarily flexible. At the same time, mito-nuclear coevolution emphasizes how organelle–nuclear interactions influence both reproductive success and the potential for reproductive isolation.

## 5. Perspectives

However, we still face three major gaps. First, cross-taxa comparisons are uneven. We need standardized imaging techniques and advanced methods like single-cell or long-read mtDNA analyses to create a comprehensive evolutionary map of mitochondrial quantity, spatial distribution, and ultrastructure. Second, the causal basis of DUI’s sex-specific “aggregation–dispersion” switch remains unclear, requiring functional dissection of receptor-mediated mitophagy, small RNAs, and M-type genomic features such as cox2 extensions and sex-linked ORFs. Third, systematic characterization of paternal mtDNA clearance in non-DUI animals could provide a framework to “reverse engineer” the suppressed or bypassed pathways in DUI.

By tackling these challenges, we can gain deeper insights into how mitochondria serve not only as powerhouses of energy but also as key players in shaping reproductive systems and diversity from an evolutionary perspective.

## Data Availability

Not applicable.
